# Study protocol: the Childhood to Adolescence Transition Study (CATS)

**DOI:** 10.1186/1471-2431-13-160

**Published:** 2013-10-08

**Authors:** Lisa K Mundy, Julian G Simmons, Nicholas B Allen, Russell M Viner, Jordana K Bayer, Timothy Olds, Jo Williams, Craig Olsson, Helena Romaniuk, Fiona Mensah, Susan M Sawyer, Louisa Degenhardt, Rosa Alati, Melissa Wake, Felice Jacka, George C Patton

**Affiliations:** 1 Murdoch Childrens Research Institute, Melbourne, Australia; 2 Centre for Adolescent Health, The Royal Children’s Hospital, Melbourne, Australia; 3 Melbourne School of Psychological Sciences, The University of Melbourne, Melbourne, Australia; 4 UCL Institute of Child Health, University College London, London, UK; 5 La Trobe University, Melbourne, Australia; 6 Health and Use of Time (HUT) Group, University of South Australia, Adelaide, Australia; 7 Deakin University, Melbourne, Australia; 8 Department of Paediatrics, The University of Melbourne, Melbourne, Australia; 9 Clinical Epidemiology and Biostatistics Unit, The Royal Children's Hospital, Melbourne, Australia; 10 University of New South Wales, Sydney, Australia; 11 Melbourne School of Population and Global Health, The University of Melbourne, Victoria, Australia; 12 School of Population Health, University of Queensland, Brisbane, Australia; 13 Centre for Youth Substance Abuse Research, University of Queensland, Brisbane, Australia; 14 Centre for Community Child Health, The Royal Children’s Hospital, Melbourne, Australia

**Keywords:** Puberty, Hormones, Adrenarche, Gonadarche, Adolescent, Cohort studies, Public health, Protocol, Epidemiology

## Abstract

**Background:**

Puberty is a multifaceted developmental process that begins in late-childhood with a cascade of endocrine changes that ultimately lead to sexual maturation and reproductive capability. The transition through puberty is marked by an increased risk for the onset of a range of health problems, particularly those related to the control of behaviour and emotion. Early onset puberty is associated with a greater risk of cancers of the reproductive tract and cardiovascular disease. Previous studies have had methodological limitations and have tended to view puberty as a unitary process, with little distinction between adrenarche, gonadarche and linear growth. The Childhood to Adolescence Transition Study (CATS) aims to prospectively examine associations between the timing and stage of the different hormonally-mediated changes, as well as the onset and course of common health and behavioural problems that emerge in the transition from childhood to adolescence. The initial focus of CATS is on adrenarche, the first hormonal process in the pubertal cascade, which begins for most children at around 8 years of age.

**Methods/Design:**

CATS is a longitudinal population-based cohort study. All Grade 3 students (8–9 years of age) from a stratified cluster sample of schools in Melbourne, Australia were invited to take part. In total, 1239 students and a parent/guardian were recruited to participate in the study. Measures are repeated annually and comprise student, parent and teacher questionnaires, and student anthropometric measurements. A saliva sample was collected from students at baseline and will be repeated at later waves, with the primary purpose of measuring hormonal indices of adrenarche and gonadarche.

**Discussion:**

CATS is uniquely placed to capture biological and phenotypic indices of the pubertal process from its earliest manifestations, together with anthropometric measures and assessment of child health and development. The cohort will provide rich detail of the development, lifestyle, external circumstances and health of children during the transition from childhood through to adolescence. Baseline associations between the hormonal measures and measures of mental health and behaviour will initially be examined cross-sectionally, and then in later waves longitudinally. CATS will make a unique contribution to the understanding of adrenarche and puberty in children’s health and development.

## Background

Puberty is a universal experience in normal human development, marking the transition from childhood to adulthood. It is accompanied by physical growth, brain maturation and sexual maturation resulting in reproductive capability [1]. The transition through puberty is also marked by an increased risk for the onset of health problems related to behaviour and emotional control [2]. The Childhood to Adolescence Transition Study (CATS) is a new longitudinal study of pubertal development that aims to prospectively examine the hormonal, psychological and social processes that may influence the onset and course of health problems between childhood and adolescence. This paper presents the research protocol for the study.

It was not until the middle of the 20^th^ century that studies began to objectively quantify puberty [3]. During the 1960s, Tanner and colleagues created a five-level staging system for external signs of pubertal development, which remains the primary system in use today [4-6]. The Tanner stages range from Stage 1, no external signs of pubertal development, to Stage 5, which indicates complete physical maturation. While Tanner described separate breast and pubic hair stages in girls and genital, testicular and pubic hair stages in boys, these are extremely highly correlated and are appropriately considered together as a single stage [7].

The onset of puberty is generally understood to be marked by progression to Tanner Stage 2, which normally occurs between 8 and 13 years in girls and about 6 to 12 months later in boys [8]. However, this view of puberty as a unitary process with a distinct onset marked by physical changes, such as hair growth, genital and skin changes or menarche, is overly simplistic. Puberty is in fact a combination of physiological processes that originate with neuroendocrinological changes several years prior to the onset of physical signs [9]. There are at least three separate neuroendocrine axes involved in the pubertal cascade; adrenarche, the activation of adrenal androgen production; gonadarche, the activation of the gonads proper, and the further activation of the growth hormone-insulin-like-growth factor (IGF) axis that occurs at puberty.

The activation of puberty is not well understood and remains under-researched. It is unlikely that a single event causes the onset of puberty but that it begins with the initiation of a complex neuroendocrine network [10]. The timing of puberty has genetic components, with nutrition, development in utero, socioeconomic factors and demographic factors in childhood also contributing [1,11].

Gonadarche, the best understood of the pubertal processes, results in sexual maturation and reproductive capability, and is physically marked by menarche in girls and spermarche in boys. It is initiated by the transition from tonic to pulsatile secretion of gonadotrophin releasing hormone (GnRH), which in turn leads to increased pituitary secretion of the gonadotrophins, follicle-stimulating hormone (FSH) and luteinising hormone (LH) [10]. Puberty as a whole is usually complete within 2–4 years following the onset of gonadarche, although time-dependent effects of sex steroids continue throughout life. However, prior to gonadarche, adrenal androgens, such as androstenedione, dehydroepiandrosterone (DHEA) and its sulphate DHEA-S begin rising, from around 6 to 8 years of age, with the maturation of the adrenal cortex in a process known as adrenarche [3].

Adrenarche is a feature of higher primate development not seen in other mammalian species apart from chimpanzees and gorillas [3]. Adrenarche is described by some researchers as 'adrenal puberty’, with the underlying physiological processes less well understood than those associated with gonadarche [12]. Adrenarche may play a role in the extension of the preadolescent phase on human ontogeny, promoting prolonged brain development of the pre-frontal cortex [13]. Adrenal androgens have a role in the development of axillary and pubic hair, the emergence of acne, particularly in females, and may play an important role in brain maturation [14]. Adrenal androgens are also precursors of the sex steroids testosterone and oestrogens [15]. It is now also clear that adrenal androgens exert direct behavioural effects and influence brain function before full reproductive maturity [16].

### Pubertal development and mental health and behaviour problems

Humans are the only animals to demonstrate major central nervous system (CNS) development at the same time as puberty. Pubertal stage, rather than chronological age, has been associated with a number of health and behaviour problems, which often persist into adulthood [17-19]. Recognising and understanding the distinction between pubertal stage (Tanner stage) and timing (onset relative to peers) is important. To illustrate, there is evidence that the timing of adrenarche and/or gonadarche might affect risk of physical and mental health problems [20,21]. The speed of transition (i.e, the tempo through puberty) may itself be linked with health and behaviour problems, although this is an area that has received little research [3,22]. The health problems associated with puberty and central to this study will be reviewed below and include:

• Mental health problems

• Antisocial behaviour and substance use

• Physical health problems and functional somatic syndromes

• Other problems such as impaired sleep and academic performance.

### Mental health problems

There is evidence that there has been a rise in adolescent mental health problems in recent decades [23]. Early puberty has been implicated in the emergence of mental health problems. Early maturing girls are more likely to have both internalising and externalising problems, while early maturing boys show higher rates of externalising symptoms [24]. Depression is more common in adult females than males, although not before puberty. This gender dimorphism emerges during puberty as a function of pubertal stage rather than age, implicating the biological changes associated with puberty [17,19,25]. Similarly, pubertal stage rather than age has been shown to be the stronger predictor of panic attack occurrence and eating disorders [18,26]. Both panic attacks and eating disorders are rare before puberty but increase dramatically in females during pubertal development [18,26]. In the past, Attention-Deficit/Hyperactivity Disorder (ADHD) was considered a childhood disorder that disappeared as children reached adolescence. However, subsequent research has demonstrated that ADHD is a chronic disorder that can persist into adolescence and adulthood [27]. To date, there have been no studies examining the role of puberty and age in ADHD onset [2]. However, there is some evidence from clinical studies that DHEA and DHEA-S are inversely correlated with ADHD symptoms [28].

### Antisocial behaviour and substance use

Puberty has been linked with antisocial behaviour in several studies. Males who enter puberty early have higher rates of antisocial behaviour during adolescence [29,30]. Pubertal stage has also been shown to be an important risk factor for antisocial behaviour. Rates of violent behaviour and aggression were found to be significantly higher in mid- and late-puberty compared with early puberty [31]. For substance use, similar patterns have also been identified. Females entering puberty early have been found to have an increased use of tobacco and alcohol in adolescence [32,33].

### Physical health problems and functional somatic syndromes

Estimates suggest that 10% of adolescents have a chronic physical health condition [34]. Pubertal timing and stage are risk factors for a number of physical health and functional somatic syndromes. To illustrate, individuals with asthma who enter puberty early are at greater risk of their condition persisting into adolescence and having increased severity in adulthood [35]. Pubertal development has also been found to be a better predictor than age of functional somatic syndromes in adolescents [36]. Like depression, many functional somatic syndromes are more common in females than males and this difference in prevalence emerges during puberty [37]. In line with this, female reproductive hormones are associated with an increased risk of migraine [38]. However, research in this area has largely been cross-sectional and so longitudinal studies are needed to examine causal pathways.

Early puberty has been linked with a number of health problems occurring later in life, such as some cancers and cardiovascular disease. Early menarche increases the risk for breast cancer leading to the suggestion that increased exposure to oestradiol and/or progesterone over time may contribute to this increased risk [39]. Similarly, girls with earlier menarche have also been shown to be at increased risk for mortality and cardiovascular disease, compared with girls maturing later, with some evidence that later puberty is protective [40].

### Other problems

Some research indicates that the timing of puberty can affect performance at school. However, these findings are limited and mixed, with some evidence suggesting children entering puberty earlier received lower grades [41] and other studies finding the opposite pattern [42]. More research examining this relationship is needed, with detailed measures of puberty. Sleep disturbance in adolescents is common [43]. Sleep problems range from insufficient sleep to more severe problems such as sleep apnoea. Sleep disturbance can have a significant impact on functioning and development, and can affect a range of factors such as academic performance and mood [43]. Nevertheless, few studies have investigated the association between sleep and psychiatric disorders in adolescents, or the effect of puberty on changes in sleep patterns and sleep disturbance. Such associations are plausible, given that sleep requirements change markedly in early adolescence at the same time as puberty, and that melatonin, a pineal hormone, which is one of the key physiological regulators of the sleep cycle, changes with puberty in healthy humans [44].

### Risk factors for early puberty

Early puberty may place an individual at risk for a variety of health and behaviour problems, and it is important to understand factors that may influence pubertal timing. A number of factors have been associated with early puberty, such as obesity, low socioeconomic status, psychosocial stress and absence of a biological father [45]. However, many of these studies view puberty as a single, discrete process, and do not differentiate between adrenarche and gonadarche. For example, menarche, an event occurring late in gonadarche, has been found to begin five months earlier in obese-overweight children compared with normal weight peers [46]. In contrast, high quality parental investment and lower marital conflict have been shown to predict later adrenarche [47]. This is consistent with life history theory, an emerging framework attempting to explain the causes and effects of individual differences in pubertal timing [48-50]. Life history theory proposes that the environment experienced during infancy and childhood (and probably prenatally) influences children’s reproductive strategies and thus, affects the timing of the transition into adolescence, marked by adrenarche [15].

### Limitations of existing research on pubertal development and health

To date, studies of pubertal development and health have been limited across various methodological areas including: a reliance on proxy measures of pubertal development; unclear and inconsistent use of definitions; cross-sectional designs; small sample sizes, and female only samples [9]. In a review of studies measuring puberty, almost a quarter of studies did not provide enough information to determine if puberty was measured by physical examination or self-report, making interpretation and replication difficult [9]. Age at menarche has been frequently used as an indicator of puberty in studies of pubertal development and mental health [19,51,52]. This marker is obviously limited to girls, occurs late in the pubertal process with almost two-thirds of girls reaching menarche in Tanner Stage 4 [53], and there is wide variability in self-reporting [9]. The first studies measuring hormones in relation to normal pubertal development were conducted during the 1980s [21,54,55]. However, studies often included participants that were too old to allow adrenarche to be studied [9]. Additionally, studies examining hormone concentrations have tended to use small sample sizes, with a few hundred participants at most, probably as a result of the cost and practicalities of collecting these samples [56-59].

Finally, the focus of previous work has been on puberty as a unitary physical process (e.g., Tanner staging) or the physical signs of gonadarche (e.g., menarche). Very little research has considered the role of adrenarche and its specific relationship with health and behaviour, or the interaction between adrenarche and gonadarche. As adrenarche and gonadarche represent different endocrine axes, it is critical to consider both processes when examining the role of puberty in health and behavioural development [3]. This failure to distinguish between adrenarche and gonadarche may contribute to the sometimes confusing and contradictory findings, which have emerged in the field.

In summary, no previous study has examined adrenarche in a large cohort and adequately examined whether differences in pubertal, social, lifestyle and biological transitions may explain the emergence of health and behaviour problems. This is despite evidence for the role of adrenarche in brain development during this period, and that adolescent onset mental and behavioural disorders have become more common in recent decades [23].

### Aims of CATS

The long-term aim of CATS is to prospectively examine how the timing and sequencing of hormonal events during puberty are associated with the onset and course of emotional, behavioural, social and learning problems through childhood and adolescence. In addition, the study will examine the influence and interaction of children’s psychological style and social context on the emergence of these problems during puberty. The first phase of this study begins in Grade 3, when children are 8–9 years of age, which will allow us to assess adrenarche. The broad research aim of this phase is to examine the associations between the onset of adrenarche and rates of emotional, behavioural and social problems. Our initial specific aims for the first phase of the study are:

1. To describe adrenal hormones and pubertal development in relation to adrenarche in 8–9 year old children.

2. To examine correlates, including both early life and current correlates, of the variation of the timing of adrenarche with a particular focus on anthropometry and social context.

3. To examine the associations between early adrenarche and emotional, behavioural, social and learning problems in 8–9 year old children.

## Methods/design

### Overall study design

CATS is a multidisciplinary five-year longitudinal cohort study, with four waves of annual data collection currently planned. The study is conducted in metropolitan Melbourne in the state of Victoria, Australia. Recruitment took place in Grade 3 (8–9 years of age), the fourth year of formal schooling, allowing the transition into early puberty to be captured. The study has been funded by the National Health and Medical Research Council of Australia (project grant number: 1010018). CATS is based at the Murdoch Childrens Research Institute and the Centre for Adolescent Health at the Royal Children’s Hospital Melbourne, Australia. Ethics approval has been granted by the Royal Children’s Hospital Human Research Ethics Committee (#31089).

#### Piloting of methods

Feasibility work was carried out before data collection began. In total, 105 participants (64 students and 41 parents) were recruited from the Royal Children’s Hospital out-patient clinics to complete questionnaires. The questionnaires were further revised and re-piloted, along with all baseline measures, at two pilot schools with 63 students and their parents. These pilot schools were recruited outside of the sampling frame and will also be pilots for future waves of data collection.

#### Project governance

A reference group for the study has been established. This group consists of representatives from each of the education sectors (Government, Catholic, Independent) as well as representatives from the pilot schools and the Melbourne Education Research Institute, The University of Melbourne. The aim of the group is to assist in the achievement of project outcomes by promoting working partnerships with the education sector and the community. It also provides an avenue for community feedback about proposed research activities, as well as the support and networking required for the promotion and implementation of the project.

### Recruitment

Participant recruitment commenced in February 2012. Recruitment took place through primary schools, which were randomly selected without replacement from a stratified (Government, Catholic, Independent strata) cluster sample of all such schools in metropolitan Melbourne educational regions with 10 or more students enrolled in Grade 3. The metropolitan area was chosen in order to facilitate follow up assessments. Permission was granted from the Victorian Department of Education and Early Childhood Development office and the Catholic Education Office Melbourne to recruit through their schools. School principals, at all schools, provided consent for their school’s participation. If a school did not consent to take part then, where possible, a replacement school was randomly selected from the same stratum and offered participation.

**Figure 1:**
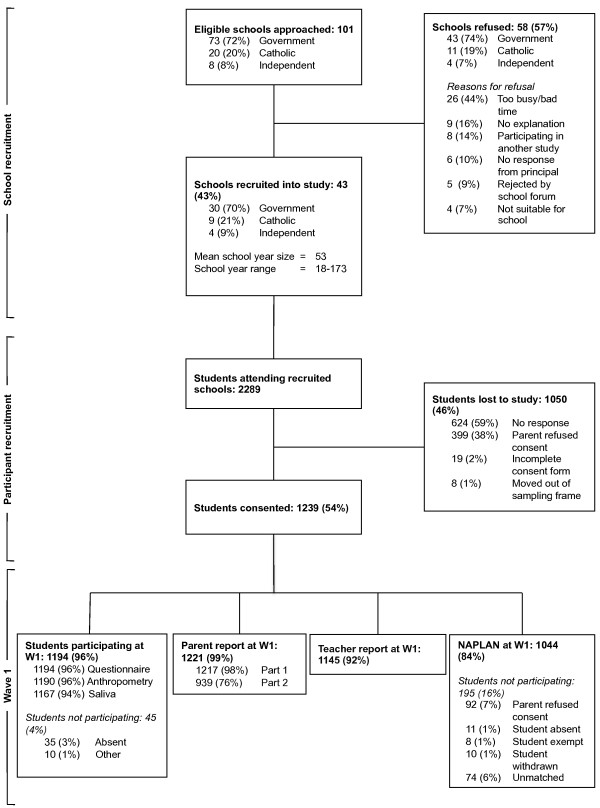
Flowchart of participants from recruitment to wave 1 data collection.

Whole year levels of participating schools were invited to take part. Information sessions for students and teachers were held at all consenting schools. A recruitment pack was given to all eligible students, which they were asked to take home to their parents/guardians. Parent consent forms were then returned to the school and collected by the research team. Every child that returned a consent form (whether accepted or declined consent) was given a small prize. The class in each school that returned the highest proportion of parent consent forms (both accepted and declined consent) was given a small prize. A total of 101 schools were approached to take part of which 43 (43%) schools agreed to participate. In total 2289 students were enrolled at these schools of which 1239 (54%) students and their parents agreed to participate. Of the students and parents who agreed to participate, 1194 (96%) students and 1221 (99%) parents took part in wave 1 data collection. Figure 1 summarises recruitment through to wave 1 data collection.

#### Sample maintenance

To assist with follow up, parents were asked to provide contact details of two additional friends or relatives. These contacts will be used to help trace participants at future waves if necessary. In an effort to maintain contact with CATS participants, a thank you card, a newsletter and a 'change of address’ form were sent after wave 1 and will be sent annually to participants. A short documentary has also been created for the study with the primary aim of enhancing participant engagement and this can be viewed on the study website [60].

### Data collection

Data collection is conducted annually. Wave 1 took place in 2012 and wave 2 is taking place in 2013. The current series of data collection is due to end in 2015. In this paper, wave 1 will be described in detail noting any changes planned for later waves. Data are collected using parent, teacher and student self-report questionnaires. Additionally, students provide a saliva sample and take part in anthropometric assessments.

#### Student

Student data collection is conducted in school during school hours. Students complete questionnaires annually and an iPad app is used to administer the questionnaires. The application works offline and so can be used in areas with no internet connection. Data are stored on the device and uploaded subsequently with connection to the internet. The application has a branched algorithmic structure allowing questions to be presented in a clear and novel format. The student questionnaire is completed in a class setting with a research assistant (RA) reading out the questions, following a standard script. This format helps students with lower literacy levels and is repeated at wave 2. At later waves students will complete the questionnaire on their own and will be asked to indicate to a supervising RA if they have questions or comprehension difficulties. At wave 1, the questionnaire took approximately 20 minutes to complete and at later waves will take no more than 45 minutes to complete.

Anthropometric measurements (height, weight, waist circumference) are completed annually. A trained RA conducts the assessment with participants, one at a time, in a private area. A saliva sample is collected at waves 1 and 3. The saliva sample is collected in the class setting using the passive drool method. Participants are asked to think of their favourite food and drool through a plastic drinking straw into a large vial. This is timed for 3 minutes and the total saliva sample provided is approximately 3 ml. Students who are unable to provide 1 ml are invited to provide a second sample and this sample is provided in a small group setting.

At wave 1, if 3 or more students were absent a second data collection session was scheduled if convenient with the school. At later waves, if students miss these sessions or have left participating schools, alternative arrangements will be made. Families will either be offered home visits, visits will be made to the new school, or they will be invited to the Royal Children’s Hospital, or to a dedicated assessment session in local areas. If families have moved outside of metropolitan Melbourne, they will be sent questionnaires and a protocol for parent collection of the anthropometric measurements.

#### Parent or guardian

A parent or guardian completes a questionnaire annually. At wave 1, there were two parent questionnaires (part 1 and part 2). Part 1 was a short questionnaire, which took no more than 10 minutes to complete and included the Strengths and Difficulties Questionnaire (SDQ). Part 1 was included with the recruitment pack and parents were asked to return it with the consent form. Part 2 was a longer questionnaire containing demographic questions and took between 20–30 minutes to complete. At later waves the questionnaires are combined into one questionnaire but with fewer demographic questions and so will take around 15 minutes to complete. The questionnaires are available in two formats: online and as a paper copy. Parent questionnaires are sent regardless of whether the child participated in a data collection session.

#### Teacher

The class teachers of all participating students are invited to complete a brief questionnaire at each wave of data collection. This is a very short questionnaire, taking about 2 minutes to complete per student and asks questions about the student’s overall academic ability, absences from school, and an overview of the student’s behavioural and emotional functioning.

### Measures

The following section describes the main measures used in CATS at every wave (unless otherwise specified). A summary of measures and the waves at which they are collected is presented in Table 1.

**Table 1:** Measures and time points in CATS

**Construct**	**Measures**	**Source**	**Wave (Age of participants in years)**			
			**1 (8–9)**	**2 (9–10)**	**3 (10–11)**	**4 (11–12)**
Pubertal development	PDS	Parent	✓	✓		
		Child			✓	✓
	SMS	Child			✓	✓
	DHEA	Child	✓		✓	
	DHEA-S	Child	✓		✓	
	Testosterone	Child	✓		✓	
Mental health & behaviour problems	SDQ	Parent	✓	✓	✓	✓
	SMFQ	Child	✓	✓	✓	✓
	SCAS	Child	✓	✓	✓	✓
	ADHD Rating Scale IV	Parent	✓	✓	✓	✓
	CDRS	Parent	✓	✓	✓	✓
	PedsQL General Wellbeing Scale	Child	✓	✓	✓	✓
	KEDS Body Image Silhouettes	Child	✓	✓	✓	✓
	Substance use	Child	✓	✓	✓	✓
Peer & family relationships	GBQ	Child	✓	✓	✓	✓
	Family relationships	Child	✓	✓	✓	✓
Physical health, nutrition & other problems	Functional somatic symptoms	Child	✓	✓	✓	✓
	Dietary Screening Tool	Parent	✓	✓		
		Child			✓	✓
	Child sleep habits	Parent	✓	✓	✓	✓
	Media engagement	Child	✓	✓	✓	✓
	Physical activity	Parent	✓	✓		
		Child			✓	✓
Anthropometry	Height	Child	✓	✓	✓	✓
	Weight	Child	✓	✓	✓	✓
	Waist circumference	Child	✓	✓	✓	✓
Academic achievement	NAPLAN	Data provided by VCAA	✓		✓	
	Global ratings of English and Maths	Teacher	✓	✓	✓	✓
Prenatal & postnatal factors	Questions assessing birth weight, gestational age, medication, substance use, and major medical conditions during pregnancy, mode of delivery, intensive care following birth, postnatal depression and breastfeeding	Parent	✓			
Parent mental health	PHQ-2	Parent	✓	✓	✓	✓
Demographics	Questions include family composition, parental education and age, annual household income, language spoken at home, ethnicity and adoption	Parent	✓			

#### Pubertal development

*Pubertal Development Scale (PDS)* – parent report at waves 1 and 2 and self-report from waves 3 onwards. This is a measure of pubertal status, which has been shown to be acceptable for use in studies of community samples of children and adolescents [61].

*Sexual Maturation Scale (SMS)* – self-report from wave 3 onwards. This is a pictorial measure used to assess pubertal stage, which has been shown to have good correlation with self-reported Tanner and physician examination [62,63].

*Saliva sample* - The primary purpose of the saliva sample is to measure hormonal indices of puberty. DHEA and DHEA-S are measured in girls and boys as an indicator of adrenarche. Testosterone is measured in both boys and girls and provides an index of gonadarche in boys. Samples are assayed using highly sensitive enzyme immunoassays. Parent consent has been obtained from 85% (n = 1057) of the cohort to store the sample for use in future, related studies. For these participants, a DNA pellet has been extracted from the sample and biobanked along with the supernatant.

#### Mental health and behaviour problems

*Strengths and Difficulties Questionnaire (SDQ)* – parent version. This is a 25-item validated measure of behavioural and emotional problems for children aged 4 to 16 years. There are 5 subscales: emotional symptoms; conduct problems; hyperactivity/inattention; peer relationship problems, and prosocial behaviour. A total problems score is derived from the first 4 subscales [64].

*Short Mood and Feelings Questionnaire (SMFQ)* – self-report. The SMFQ is used to measure depressive symptoms. The short form has been validated for use in community samples with children as young as 7 years of age [65,66]. A subset of items is used at waves 1 and 2.

*Spence Children’s Anxiety Scale (SCAS)* – self-report. An adaptation of the SCAS is used to measure anxiety symptoms [67].

*ADHD Rating Scale IV* – parent version. This is an 18-item validated scale measuring the core symptoms of ADHD that is directly linked to DSM-IV diagnostic criteria for ADHD [68].

*Conduct Disorder Rating Scale (CDRS)* – parent version. A validated measure of conduct disorder for children aged 5 to 12 years [69], with a subset of items used at waves 1 and 2.

*PedsQL General Wellbeing Scale* – self-report. This is a widely used brief measure of health-related quality of life [70,71]. A subset of items is used at waves 1 and 2.

*Kids Eating Disorder Survey (KEDS) Body Image Silhouettes* – self-report. This measure is a valid and reliable tool for assessing body image in children [72,73].

*Substance use* – self-report. Single items on alcohol and tobacco use were adapted from Monitoring the Future and CDC Youth Risk behavior survey, as used in the International Youth Development Study (IYDS) [74-76].

#### Peer and family relationships

*Gatehouse Bullying Questionnaire (GBQ)* – self-report. The GBQ is a short and reliable tool for measuring the occurrence of bullying in schools [77], with a subset of items used at waves 1 and 2.

*Family relationships*– self report. Items were adapted from those used in IYDS on family conflict, management and attachment [74-76].

#### Physical health, nutrition & other problems

*Functional somatic syndromes* – self report. Pain and symptoms questions were derived from the SCL-90 symptoms checklist [78] together with a pain manikin [79]. Pain manikins have been shown to be acceptable alternatives for written questions [80].

*Dietary Screening Tool* – parent report. This is a brief and practical set of questions designed to assess diet quality.

*Child sleep habits* – parent report. Items used in the Longitudinal Study of Australian Children (LSAC) assess child bed and wake time, as well as any regular problems and parent concerns.

*Media engagement* – self report. Items were adapted from LSAC regarding access to media at home and the Lodz Electronic Aggression Questionnaire (LEAPQ) scale [81].

*Physical activity* – parent report. Questions on hours spent in sedentary activity and participation in sports were adapted from LSAC.

#### Anthropometry

*Height* - at each assessment, student height is measured to the nearest 0.1 cm using a portable rigid Invicta stadiometer. Height is transformed to z-scores based upon age and gender related reference charts [82].

*Weight* – at each assessment, student weight is measured to the nearest 0.1 kg using regularly calibrated Tantia THD 646 digital scales. Students are asked to remove shoes, coats and heavy clothing items. Body Mass Index (kg/m^2^) is calculated and transformed, along with student weight, to z-scores based upon age and gender related reference charts [82].

*Waist circumference* - is assessed with a non-stretch anthropometric tape according to the International Society for the Advancement of Kinanthropometry (ISAK) protocols. Waist circumference is an acceptable, non-invasive and reliable indicator of intra-abdominal fat and is measured to the nearest 0.1 cm [83].

All RAs are trained by an experienced measurer. Accuracy and precision are quantified using Technical Error of Measurement (TEM). Before being allowed to act as a measurer, the RA must demonstrate inter-tester TEMs of ≤2% and intra-tester TEMs of ≤1.5% [84].

#### Academic achievement and school achievements

*National Assessment Programme – Literacy And Numeracy (NAPLAN)* - parental consent has been obtained from 93% (n = 1147) of the cohort to obtain students’ NAPLAN results. NAPLAN is a nationwide programme conducted in Grades 3, 5, 7 and 9, which assesses the following domains: reading; writing; spelling; grammar and punctuation, and numeracy. A scaled score and a band score are provided for each domain completed by each child. There are ten bands per domain and this measure describes student achievement. The same bands are used across school grades. CATS will obtain students’ NAPLAN results at waves 1 and 3. The data will be provided by the Victorian Curriculum and Assessment Authority (VCAA).

*Global ratings of English and Maths* - teacher report. Items were adapted from those used in LSAC.

#### Prenatal and postnatal factors

Questions about the prenatal and postnatal environment were included in the wave 1 parent questionnaire. These covered birth weight, gestational age, medication, substance use, and major medical conditions during pregnancy, as well as mode of delivery, intensive care following birth, postnatal depression and breastfeeding.

#### Parent mental health

*Patient Health Questionnaire-2 (PHQ-2)* – parent self-report. This is a 2-item questionnaire validated as a screening test for depressive disorder [85,86].

#### Demographics

Demographic questions were included in the wave 1 parent questionnaire. These covered family composition, parental education and age, annual household income, language spoken at home, ethnicity and adoption.

### Sample size

It is estimated that 16.1% of girls and 6.3% of boys will have definite indication of pubertal onset (adrenarche) at 8–9 years old [87]. Expecting 134 children with early puberty and 1060 children not experiencing early puberty, the cohort would have 88% power to detect a minimum difference of 0.3 standard deviations (or 82% power to detect a minimum difference of 0.275 standard deviations) in emotional, social, behavioural or learning problems in the baseline comparisons. These estimates allow for clustering within school classes, assuming a mean cluster size of 10 children participating from each class and an intra-class correlation of 0.01 reflecting a modest degree of similarity in outcomes for children in the same school class.

The power calculation for the longitudinal analysis was based on the incidence of one primary outcome, high depressive symptoms. In our previous work in 10–15 year olds, 12% of boys and 19% of girls had incident episodes of high depressive symptoms over a 2-year follow up with a doubling in prevalence in girls between 10 and 15 years [25]. We anticipate low attrition of 1% per annum based on our previous longitudinal studies [88]. Assuming that over 4 years of data collection we have an incidence of 15% in boys and 20% in girls, we would be able to detect a minimum odds ratio (OR) of 1.7 with 85% power (or a minimum odds ratio (OR) of 1.8 with 91% power) for an exposure with 30% prevalence (e.g., earlier versus later puberty) allowing for sample attrition and clustering by school class with an intra-class correlation of 0.01.

### Data analysis

During the first phase of the study, the adrenal hormones and pubertal development stage will be described. Percentile distributions for DHEA and DHEA-S and percentage of children in each category of PDS will be presented.

Modelling framework will be used to examine the relationship between early life and current correlates with adrenal androgen levels. DHEA and DHEA-S measures will be log transformed prior to analysis to achieve normality. Tobit models will be used to handle the left censoring of the undetectable adrenal measures. The adrenal androgens levels are thought to be independent, with no correlation between student measures within schools. Multilevel Tobit models will be used to investigate this assumption. These models will focus on looking at the linear and non-linear relationships between anthropometric measures (e.g., BMI and waist circumference) and the effects of earlier measures (e.g., birth weight and family structure) on adrenal androgen levels. The effect of these factors will be estimated in separate models, first unadjusted and then adjusted for potential confounders such as age, diet and exercise.

GEE modelling framework will be used to investigate the associations between early adrenarche and emotional, behavioural, social and learning problems in 8- to 9-year-olds. This approach will allow for the potential correlation between the outcome measures of children in the same class to be taken into account. Linear regression will be used to analyse continuous measures and logistic regression will be used to analyse binary outcomes. The effect of early adrenarche on the outcome measures will be estimated unadjusted and then adjusted for potential confounders, such as demographic variables, including age. Where appropriate, analyses will be stratified by gender.

The complete cohort will provide rich detail of the development, lifestyle, external circumstances and health of these children over their transition from childhood through to adolescence. We envisage several detailed analyses to investigate the associations between these factors over time. Our initial analysis will focus on an examination of early puberty and mental health. In particular, we will use discrete time event history models to establish if early puberty is related to the onset of depressive symptoms in adolescence [89]. These models will allow us to investigate if children with early puberty are at risk for experiencing depressive symptoms earlier than those who experience puberty later. The repeated measures modelling framework will also allow us to investigate the role of other factors and to examine if these effects change over time.

## Discussion

Puberty marks the transition from childhood to adolescence and yet despite the clearly established increased risk for the onset of health problems related to behaviour and emotional control during this transition [2], there is limited previous research, which has been hindered by a number of important methodological weaknesses. CATS will be one of the first community-based prospective, longitudinal studies, examining the factors that influence the onset and course of health problems that begin during puberty.

One of the unique strengths of CATS is that data collection begins with children aged 8–9 years. Most previous studies of puberty have focused on students in secondary school, when most are well into the pubertal process. CATS will capture the transition into the earliest phase of puberty and will allow the process of adrenarche and its association with behavioural and emotional problems to be explored. In later years, gonadarche and the IGF axis will become the focus. CATS will offer a unique opportunity to examine adrenarche, gonadarche and the pubertal growth spurt, and their relationships and associations with health and behaviour in the same cohort of children. Another important strength of CATS is that it is one of few studies world-wide collecting biological measures as well as anthropometric measurements and questionnaire data from multi-informants. Previous studies have relied on self- or parent-report of pubertal stage, which are relatively insensitive measures and correlate only weakly with pubertal hormones [9]. We are not aware of a larger cohort collecting hormonal measures in children of this age.

Mental and behavioural disorders are one of the largest contributors to disease burden across adolescence and young adulthood and represent a major public health problem [90]. Once established, adolescent onset mental and behavioural disorders have a high likelihood of persistence into adult life and contribute to adult mental health disorders, cardiovascular disease and cancer, as well as to social disadvantage. Compared with other phases of development (e.g., the first three years of life and later adolescence), puberty and its disorders remain unexplored and poorly understood. This study has the potential to profoundly change our understanding of pubertal risk processes and to identify potential interventions. The findings will be relevant to families, as well as medical, mental health and education professionals, and policy makers.

A number of additions to the study are underway or planned. For example, iCATS (i.e., Imaging brain development in the Childhood to Adolescence Transition Study) is a neuroimaging project embedded within CATS and funded by the Australian Research Council (DP 120101402). iCATS aims to examine the associations between the timing of puberty and brain structure and function in a subsample of 120 participants who took part in wave 1.

We also plan to undertake a study of epigenetic changes associated with puberty. This work will have three main aims: 1) investigating early life epigenetics that may be linked with early pubertal onset; 2) exploring epigenetic changes that might occur across puberty in response to ongoing stressors, and 3) examining associations between epigenetic markers during early puberty and health and behaviour outcomes. Ideally, CATS aims to establish the foundations for continued data collection into secondary school and beyond, allowing the relationship between adrenarche and gonadarche and the emergence of health and behaviour problems to be explored into adolescence and adulthood.

## Abbreviations

ADHD: Attention-Deficit/Hyperactivity Disorder; BMI: Body Mass Index; CATS: Childhood to Adolescence Transition Study; CDRS: Conduct Disorder Rating Scale; CNS: Central nervous system; DHEA: Dehydroepiandrosterone; DHEA-S: Dehydroepiandrosterone sulphate; FSH: Follicle-stimulating hormone; GBQ: Gatehouse Bullying Questionnaire; GH: Growth hormone; GnRH: Gonadotrophin releasing hormone; IGF: Insulin-like growth factor; ISAK: International Society for the Advancement of Kinanthropometry; KEDS: Kids Eating Disorder Survey; LH: Luteinising hormone; LSAC: Longitudinal Study of Australian Children; NAPLAN: National Assessment Programme – Literacy and Numeracy; NHMRC: National Health and Medical Research Council; PDS: Pubertal Development Scale; PHQ-2: Patient Health Questionnaire-2; RA: Research Assistant; SCAS: Spence Children’s Anxiety Scale; SDQ: Strengths and Difficulties Questionnaire; SMFQ: Short Mood and Feelings Questionnaire; SMS: Sexual Maturation Scale; TEM: Technical error of measurement; VCAA: Victorian Curriculum and Assessment Authority.

## Competing interests

The authors declare that they have no competing interests.

## Authors’ contributions

GP, NA, RV, JB, TO, JW, CO, SS, LD, RA, MW, FJ and FM contributed to the overall design and conception of the study and assisted with the writing of the grant application. LM, JS, GP and NA drafted and revised this manuscript. LM, GP and NA contributed to study implementation and coordination. FM and HR contributed to the statistical design of the study. All authors read and approved the final manuscript.

## Pre-publication history

The pre-publication history for this paper can be accessed here:

http://www.biomedcentral.com/1471-2431/13/160/prepub
